# Beyond Inflammation: Sarcopenia and Disease Phenotype Predict Unfavourable Course in Crohn’s Disease—A Retrospective Observational Study

**DOI:** 10.3390/biomedicines14081855

**Published:** 2026-08-18

**Authors:** Vasile-Claudiu Mihai, Marius Lucian Savin, Ioana-Irina Rezuș, Vasile-Lucian Boiculese, Alina Ecaterina Jucan, Georgiana Sârbu, Carmen Atodiresei, Mihaela Dranga, Otilia Nedelciuc, Liliana Gheorghe, Cristina Cijevschi-Prelipcean, Cătălina Mihai

**Affiliations:** 1 Grigore T. Popa University of Medicine and Pharmacy, 700115 Iasi, Romania; 2 ‘Sf. Spiridon’ Emergency Clinical County Hospital, 700111 Iasi, Romania; 3 Arcadia Medical Hospital, 700620 Iasi, Romania

**Keywords:** inflammation, sarcopenia, inflammatory bowel diseases, muscle, nutritional status

## Abstract

**Introduction**: Chronic inflammation in Crohn’s disease (CD) is associated with systemic alterations, including the loss of skeletal muscle mass. While nutritional deficits are known to impact clinical trajectories, the specific prognostic value of muscle wasting remains underutilised. The primary objective of this study is to construct a composite score integrating sarcopenia with paraclinical parameters to predict an unfavourable disease outcome (UO) more accurately. **Materials and Methods**: We conducted a retrospective study of patients with CD who were hospitalized at our institution between January 2020 and June 2024 and were treated with biologics or small molecules. Using previous CT scans, we measured skeletal muscle area (SMA) and skeletal muscle index (SMI) to assess their association with disease outcomes. Univariate and multivariate analyses were performed to identify significant predictors of UO, and Area Under the Curve (AUC) values were calculated to assess the predictive performance and to construct a composite prognostic score. **Results**: Multivariate analysis in the study group (52 patients) identified sarcopenia (low SMI), faecal calprotectin levels >129 μg/g, and complicated disease behaviour (Montreal B2/B3) as independent predictors of UO. Integrating these parameters into a composite risk score resulted in a promising predictive model with an Area Under the Curve (AUROC) of 0.864, significantly outperforming individual biomarkers. **Conclusions**: We performed a hypothesis-generating study suggesting that nutritional assessment in CD could provide an important benefit in stratifying patients for whom additional nutritional interventions may enhance the long-term outcomes.

## 1. Introduction

Sarcopenia is a clinical condition defined as a progressive and generalised loss of muscle mass and function [1]. Age-related sarcopenia is relatively frequent, with an incidence of up to 29% in older persons [2]. Still, various pathologies may accentuate this process in younger patients and lead to a vicious cycle of auto-aggravation [3]. Mechanisms implicated in the imbalance between muscle production and muscle wasting include chronic inflammation, malabsorption, metabolic imbalances, and high protein intake to sustain vital visceral functions. Loss of muscle mass is associated with an increased likelihood of disease evolution, complications, and high hospitalization costs [4,5,6].

Several tools for defining sarcopenia are available and focus on three aspects: muscle mass, muscle strength, and physical ability. These tools include patient-reported questionnaires (SARC-F [7]), muscle strength measurements through physical tests (dynamometry, the chair stand test), general physical performance, and muscle quality determinations. The latter can be achieved by calculating multiple body composition values based on dual X-ray absorptiometry (DXA), Computed Tomography (CT), and Magnetic Resonance Imaging (MRI), and bioelectrical impedance analysis (BIA) [8].

Inflammatory bowel diseases (IBDs) are disorders associated with chronic or relapsing inflammation of the gastrointestinal tract, encompassing two principal patterns of distribution: Crohn’s disease (CD) and ulcerative colitis (UC) [9]. Multiple mechanisms are implied in the pathophysiology of IBD-associated sarcopenia in varying degrees, among which the most important are malabsorption, chronic inflammatory status, vitamin deficiency, and imbalances in the muscle–gut axis [5]. The mechanisms involved in the pathogenesis of IBD-associated sarcopenia are more prominent in CD than UC [3,10]. Some articles state that up to 52% of patients with CD may experience sarcopenia during their disease evolution [11].

The natural evolution of CD is highly variable, but most of the patients exhibit a recurrent and remissive pattern, with up to 20% of patients experiencing yearly relapse [12]. Disease flares are highly unpredictable, and no study has managed to find the ideal prognostic tool for recurrence [13]. This significantly impacts the quality of life of CD patients, suggesting the need for a continuous search for predictive factors that can be intervened upon and reduce the rate of recurrence or delay the appearance of new flares.

Sarcopenia is commonly assessed via CT or MRI using the total psoas area and skeletal muscle area (SMA) at the level of the L3 vertebra, considered the gold standard [14]. Skeletal muscle index (SMI), defined as the ratio of SMA to patient height, may be a more appropriate height-normalised measure. There is no consensus on cut-off values for defining sarcopenia using the SMI, with different values reported in the literature due to population and individual variability [15]. Some studies demonstrated that sarcopenia is a risk factor for postoperative complications [16,17], complicated disease, higher values of inflammatory markers, and loss of response (LOR) to treatment [9], while others showed no significant predictive value for treatment and surgical decisions [18].

Regarding inflammatory status, a wide array of serological and stool pro- and anti-inflammatory molecules have been measured in IBD patients (e.g., IL-6, IL-8, TNF- α, C-reactive protein (CRP), faecal calprotectin) with disparate results. Of these, only faecal calprotectin and CRP are routinely used for disease monitoring, with sensitivities for predicting recurrence ranging from 46 to 95% depending on the cut-off values and study conditions [12,13,19].

Although these studies focused on determining the prognostic capacity of singular inflammatory or nutritional parameters for disease relapse, we presumed that a composite scoring system would likely maximise the predictive value of these variables. The aim of our study is to determine whether additional focus should be placed on the combined assessment of patients’ nutritional and inflammatory status rather than on univariate analysis.

## 2. Materials and Methods

### 2.1. Study Design and Patients

Our study was conducted in accordance with the Declaration of Helsinki and reported following the recommendations of the STROBE checklist (see Supplementary Table S1). Our study was approved by the Research Ethics Committee of the “Grigore T. Popa” University of Medicine and Pharmacy, Iași (Project identification code 539/15.02.2025). Informed consent was waived due to the retrospective nature of the study.

This research was funded by The Health Programme (PS) 2021–2027, Policy Objective 4, Priority 3, Project title “Support for Enhancing Performance and Innovation in Excellent Medical Doctoral Research”, acronym INNODOC-MED, SMIS code 351058.

This retrospective observational study included patients with CD followed up in the Gastroenterology Department of the “Sf. Spiridon” Emergency Clinical County Hospital, Iași, Romania.

We included patients with a confirmed diagnosis of CD who were hospitalised in our department between January 2020 and June 2024. The inclusion criteria were: adult patients with confirmed CD according to the ECCO-ESGAR Guideline for Diagnostic Assessment of IBD [20] undergoing biologic or small-molecule treatment, in whom a CT examination and blood tests were performed in the same week and were available for data collection. We included only patients who had at least one follow-up visit between 6 and 12 months after baseline imaging. The exclusion criteria included: patients under 18 years; lack of imaging or blood test data; corticoid therapy; lack of sequential follow-up visits; other chronic diseases that may lead to patient wasting (e.g., malignancies, congestive heart failure, chronic kidney disease, neuromuscular disease); and pregnancy. As this was a retrospective observational study, no formal a priori size calculation was performed. All consecutive patients who met the eligibility criteria during the predefined period were included.

Demographic, clinical, and laboratory data were extracted from medical records, with a minimum follow-up of one year. Baseline and subsequent CT or MRI scans were analysed to evaluate disease status.

### 2.2. Data Collection

CT image acquisition was performed at our hospital using a Phillips Incisive scanner (2020, Koninklijke Philips N.V., Amsterdam, Netherlands, 64 slices). Scanning protocols included the entire abdomen and pelvis with a standardised multiphase protocol, including a native scan and bolus-tracker post-contrast arterial and venous scan. No bowel preparation was performed. A clinician prescribed imaging to evaluate suspected disease complications, assess disease involvement and severity, and evaluate unrelated intercurrent conditions. Two radiologists independently evaluated the images to extract the target parameters, and the mean value was included in the statistical analysis. Intraclass correlation coefficient (ICC) was calculated using a two-way random-effects model for absolute agreement.

Data were extracted from medical records using a standardised collection sheet. Recorded variables included demographics, disease phenotype, smoking status, and present and past therapeutic regimens. Additionally, routine laboratory parameters were analysed to evaluate nutritional status (haemoglobin and albumin) and the inflammatory burden (CRP and faecal calprotectin).

### 2.3. CT-Based Body Composition Analysis

The SMA and SMI at the level of the L3 vertebral body were extracted from the available CT images using 3D Slicer (v5.8.1), a free, open-source, extensible software application for medical image computing and visualization [21]. We measured the total area of skeletal muscle tissue on a single slice at the level of the L3 vertebra (which included the transverse processes) by selecting the range of densities specific for muscle tissue (between −29 to +150 Hounsfield units—HU) and excluding the pixels in range that were represented by other solid structures with the same HU values. The segmentation tool of 3D Slicer was used to calculate the pixel area automatically. The SMA was normalised for height by dividing it by the square of height (in meters), yielding the SMI (cm^2^/m^2^).

In addition to these metrics (SMA and SMI), we also analysed the inflammatory markers habitually evaluated in CD (CRP, calprotectin), as data suggests that chronic inflammation and malabsorption drive muscle wasting (sarcopenia). Laboratory indices such as albumin and haemoglobin offer a systemic overview of the effects of chronic inflammation on metabolism, hinting at poor physiological reserve and association with adverse outcomes. Finally, behavioural parameters, such as the Montreal disease phenotype (stricturing/penetrating disease) and a history of therapeutic failure, were included to assess cumulative structural damage and the disease’s inherent refractoriness.

Aiming to increase our sensitivity and reduce bias, we performed a multiple logistic regression model on the significant parameters identified in our univariate analysis. We assumed that a holistic evaluation of the patient’s status could better capture the interplay of different factors, resulting in improved predictive power.

### 2.4. Outcome Measures and Statistical Analysis

The primary outcome of our study was the rate of unfavourable disease evolution despite correct therapeutic prescription and administration. Loss of response to a therapeutic drug is a complex concept that requires integrating clinical, endoscopic, and/or biologic analyses of inflammatory status, and there is no international consensus on its definition [22]. Regular follow-up consisted of anamnesis and routine biological tests performed at every treatment session. According to the treatment regimen and the hospital follow-up protocol, a clinical evaluation was performed every 8 to 12 weeks, with blood and stool tests every 3 to 6 months. Subsequent endoscopy was performed either due to relapse or at 9 to 13 months post treatment induction, while CT scans interval from baseline varied between 3 and 15 months in the selected cohort. After this interval, repeat imaging and endoscopy showed a greater variability. No significant differences regarding follow-up between the outcome groups were identified.

Based on the data obtained from patient medical records, we created two outcome groups:

- Patients with sustained clinical (according to the Crohn’s disease activity index—CDAI) and biologic (normalization or reduction by >50% of the initial CRP value +/− calprotectin levels when available) remission at the follow-up visits, in whom the colonoscopy and/or imaging (either CT or MRI) showed no acute bowel inflammation were considered sustained responders and added to the favourable outcome group (FOG);
- Patients in whom the disease evolution was marked by relapse, the need for changing the therapeutic line or addition of corticotherapy, surgery, and endoscopic and/or imaging findings suggestive of active disease, were included in the unfavourable outcome group after ruling out other conditions that may simulate active CD (UOG).

Statistical analysis was performed using IBM SPSS Statistics, version 27.0 (IBM Corp., Armonk, NY, USA). Data distribution normality was assessed using the Shapiro–Wilk test. Continuous variables were expressed as mean ± standard deviation (SD) for normally distributed data, or as median with interquartile range (IQR) for non-normally distributed data. Categorical variables were presented as frequencies and percentages.

To determine the association between study variables and disease outcomes (favourable versus unfavourable disease evolution), we used parametric tests (independent-samples *t*-test) for normally distributed continuous variables and nonparametric tests (Mann–Whitney U test) for those with nonnormal distributions. Categorical variables were compared using the Chi-square test or Fisher’s exact test, as appropriate. Bivariate analysis was conducted to assess correlations between physiological parameters (e.g., SMM and SMI) using Pearson or Spearman analysis as appropriate.

Univariate and multiple binary logistic regression analyses were performed to identify independent predictors of UO. Finally, Receiver Operating Characteristic (ROC) curve analysis was used to calculate the Area Under the Curve (AUC) and to determine optimal cut-off values for constructing the predictive scoring system. A *p*-value of <0.05 was considered statistically significant. The Hosmer–Lemeshow goodness-to-fit test was performed to assess the calibration of the proposed prediction model (with a *p*-value > 0.05 indicating adequate model fit).

Internal validation was implemented to increase the confidence of our proposed score. Bootstrap analyses were performed using R [23] within the RStudio integrated development environment (version 4.6.1, 2026.06.0, Build 242).

The optimal score cutoff was determined on the original dataset using the Youden index, and receiver operating characteristic (ROC) curve analysis, including calculation of the area under the curve (AUC), sensitivity (Se), and specificity (Sp), was performed using the pROC package, version 1.19.0.1 [24]. ROC curves with corresponding bootstrap confidence bands were generated using the ggplot2 package, version 4.0.3 [25].

Internal validation of the score was conducted using the nonparametric bootstrap method, based on 1000 resamples with replacement, implemented via the boot package, which is built on the bootstrap methodology described by Davison and Hinkley [26] and implemented for R by Canty and Ripley [27]. In the first stage we applied the bootstrap method to estimate the precision of the AUC, Se and Sp measures (represented graphically with confidence interval) according to our proposed coefficients. The input variables and score coefficients were fixed.

In the second stage, we applied Harrell’s optimism-correction method [28]. Keeping the three input variables fixed, we used 1,000 bootstrap resamples to fit logistic regression models, adjust the score coefficients, determine the optimal cut-off point on the ROC curve, and calculate the AUC, Se, and Sp.

## 3. Results

### 3.1. Patient Demographics

**Figure 1:**
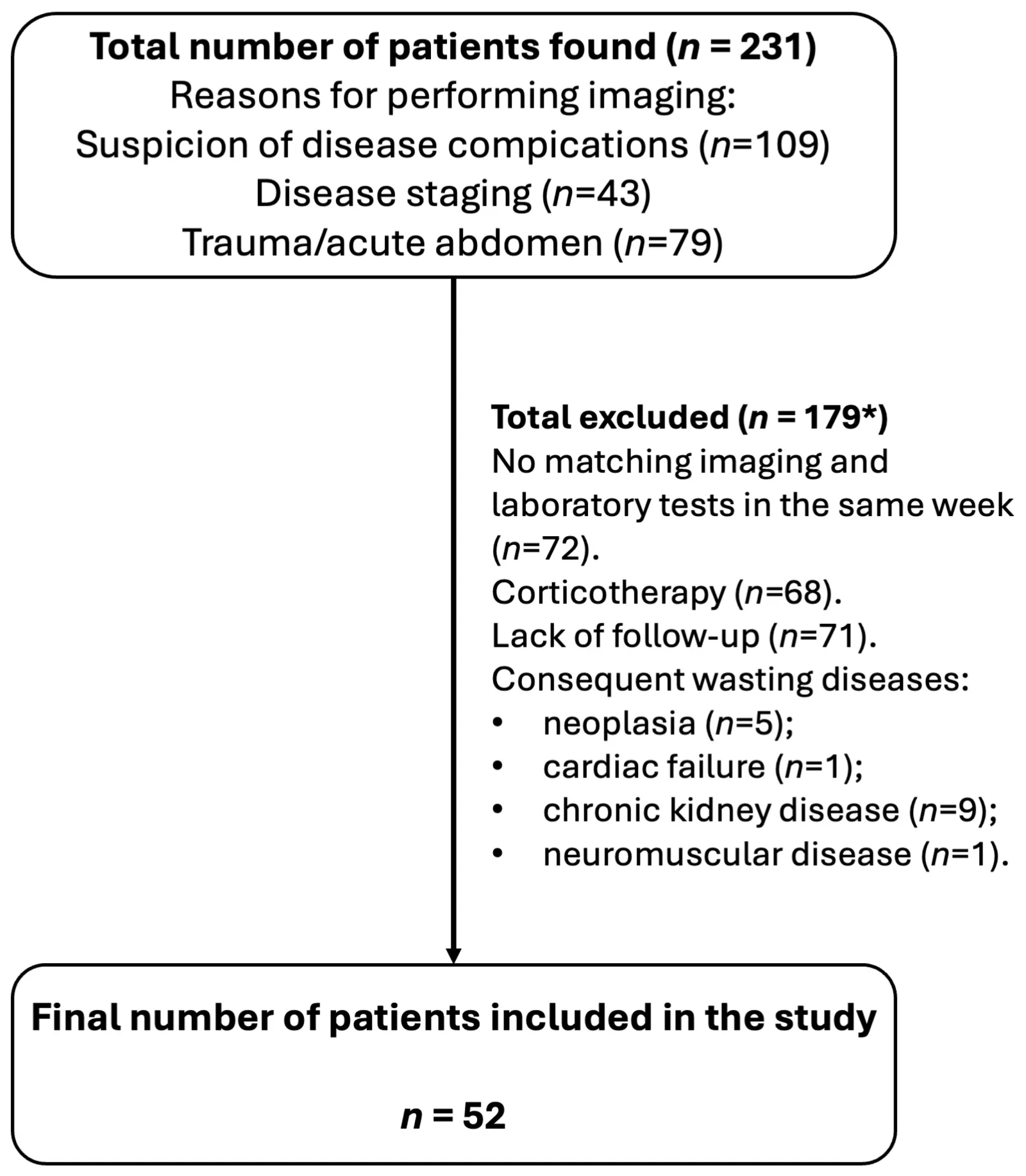
Patient selection flowchart. * Total number of patients excluded = 179. Patients with overlapping criteria = 48, of which those presenting 2 criteria = 32; 3 criteria = 15; 4 criteria = 1.

The initial search of the prospectively maintained database yielded 231 patients with CD in whom imaging was performed to evaluate suspected disease-associated complications (*n* = 109), disease staging (*n* = 43), or unrelated pathologies (*n* = 79) such as trauma or acute abdomen. After applying the exclusion criteria (Figure 1), we were left with 50 patients (52% male), with a median age of 46.49 years and a mean disease duration of 5.58 years. Patient demographics are detailed in Table 1.

In our study, only two patients declared the continuation of tobacco use despite their diagnosis; thus, we eliminated this factor from further analysis due to the low number of patients.

### 3.2. Parameter Selection and Rationale

Univariate analysis identified distinct predictors of UO across nutritional, inflammatory, and behavioural domains (Table 2). Significant values were obtained for SMA and SMI, while the binary form of sarcopenia (present/absent) according to cut-off values available in the literature was evaluated for ease of implementation. Inflammatory markers included CRP and faecal calprotectin, with the latter chosen for its high specificity to intestinal inflammation. Finally, disease behaviour, as assessed by the Montreal score, and previous treatment failure both correlated well with the UOG.

### 3.3. Nutritional Status Parameters

Among nutritional parameters, both SMA and SMI showed statistically significant associations with UO (*p* = 0.04 and *p* < 0.001, respectively). The interobserver agreement for the measurement of SMA was excellent (ICC (2, 1) = 0.984, 95% CI 0.972–0.991, *p* < 0.001). These SMA and SMI are interdependent, with a bivariate correlation analysis yielding a correlation coefficient of r = 0.908 (*p* < 0.001). The AUC for SMA was 0.596, lower than that for SMI (0.663); therefore, we proceeded to investigate SMI.

Using the Youden method, we identified SMI cutoff points for sarcopenia that closely mirrored those reported in larger cohorts, specifically those proposed by Liu et al. [6]. Therefore, we applied their standardised definitions—SMI < 32.5 cm^2^/m^2^ for women and <44.77 cm^2^/m^2^ for men—to stratify our patients.

To determine the optimal format for the predictive model, we compared the diagnostic performance of the SMI as a continuous variable with its binary categorical form (sarcopenia). The ROC curve analysis for continuous SMI yielded an AUC of 0.663, similar to the categorical form at 0.657 (95% CI = 0.485–0.838). Given the minimal loss in predictive power (0.006) and the facility of a binary scoring system, the categorical form was selected for integration into the multivariate risk score.

Conversely, lower serum albumin and haemoglobin levels did not show statistically significant correlations with the UOG (*p* = 0.672 and *p* = 0.138, respectively).

### 3.4. Inflammatory Status Parameters

**Figure 2:**
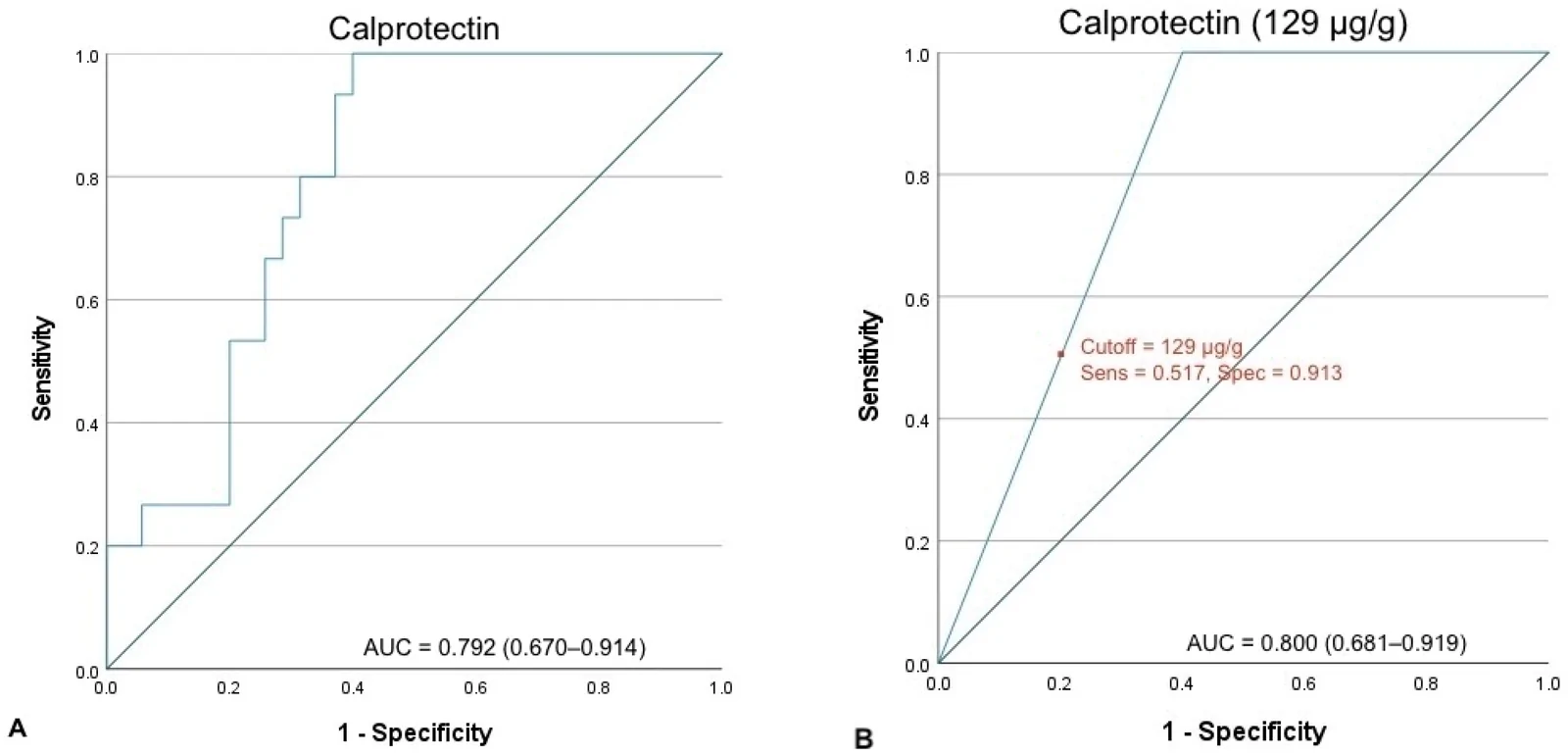
Receiver operating characteristic (ROC) curve (light blue) for the value of calprotectin without a discriminative value (**A**) and of the calprotectin threshold of 129 μg/g (**B**) in predicting disease relapse. AUC values are presented as mean (95% confidence interval). The 45° diagonal (dark green) represents the line of no discrimination (AUC = 0.5).

Regarding inflammatory burden, both CRP and faecal calprotectin were significantly elevated in the UOG (*p* = 0.02 and *p* < 0.001, respectively), suggesting that patients with more severe disease are less likely to respond to treatment. However, CRP levels are highly nonspecific and can be elevated in a wide range of infectious and inflammatory conditions. This may explain why calprotectin’s statistical power was greater, as it is specific to CD. Consequently, ROC analysis identified a faecal calprotectin level ≥129 μg/g as a significant predictor of relapse, yielding an AUC of 0.800 (95% CI 0.681–0.919) (Figure 2).

### 3.5. Disease Behaviour Parameters

We analysed behavioural parameters including disease phenotype (Montreal classification), prior surgery, and treatment history. Patients with a history of complicated disease (Montreal B2 stricturing or B3 penetrating phenotypes) (*p* = 0.003) and those with prior therapeutic failure (*p* = 0.01) were significantly more likely to experience relapse compared to those with inflammatory-only behaviour (B1) or no history of failure.

### 3.6. Statistical Selection of Predictors for the Multivariate Model

To construct a practical predictive score, variables were selected for the multiple logistic regression model based on statistical strength, independence, and clinical utility.

In the nutritional domain, both continuous (SMA and SMI) and binary (sarcopenia) parameters were significantly associated with the UOG. We prioritised sarcopenia based on SMI cut-off values due to its simplicity of use and normalisation.

Regarding inflammatory markers, both CRP and faecal calprotectin had significant correlations to an UO. Calprotectin was selected over CRP due to its higher specificity for intestinal inflammation and stronger statistical significance (*p* < 0.001 vs. *p* = 0.02). A cutoff value of >129 μg/g was established using the Youden index (J = 0.6) and included in the model as a categorical variable, as this format proved more statistically robust (*p* < 0.001) than the continuous scale form (*p* = 0.551) in regression analysis.

In terms of disease behaviour, both “previous treatment failure” and “complicated disease phenotype” (Montreal B2/B3) were significant predictors (*p* = 0.01 and *p* = 0.003, respectively). Although these variables were not strongly correlated (r = −0.07), we selected the complicated disease phenotype (B2/B3) for the final model to maintain simplicity and because it yielded a higher individual AUC (0.703) than treatment history (0.657). The decision was in accordance with the population size, as adding a fourth variable would additionally decrease the number of events per predictor variable (EPV = 5.7 in the current form).

Consequently, the binary logistic regression analysis was constructed using three independent variables representing distinct pathophysiological mechanisms: Sarcopenia (nutritional status), Calprotectin >129 μg/g (inflammatory load), and Disease Behaviour (B2/B3) (cumulative structural damage). The Hosmer–Lemeshow test showed no evidence of lack of fit (Supplementary Table S2). The resulting model proved highly effective, yielding an AUROC value of 0.864 (Table 3).

The clinical prediction score was derived from the regression coefficients of the final multiple logistic regression model. The coefficients were scaled relative to the smallest Exp(B) coefficient and rounded to practical values to obtain a simplified point-based scoring system. Points were allocated proportional to the regression Odds Ratios (Exp(B)): sarcopenia was assigned 1 point (odds ratio ≈ 4.8), faecal calprotectin >129 μg/g was assigned 1.75 points (odds-ratio ≈ 8.5), and complicated disease was assigned 2 points (odds-ratio ≈ 10.3) (Table 4).

**Figure 3:**
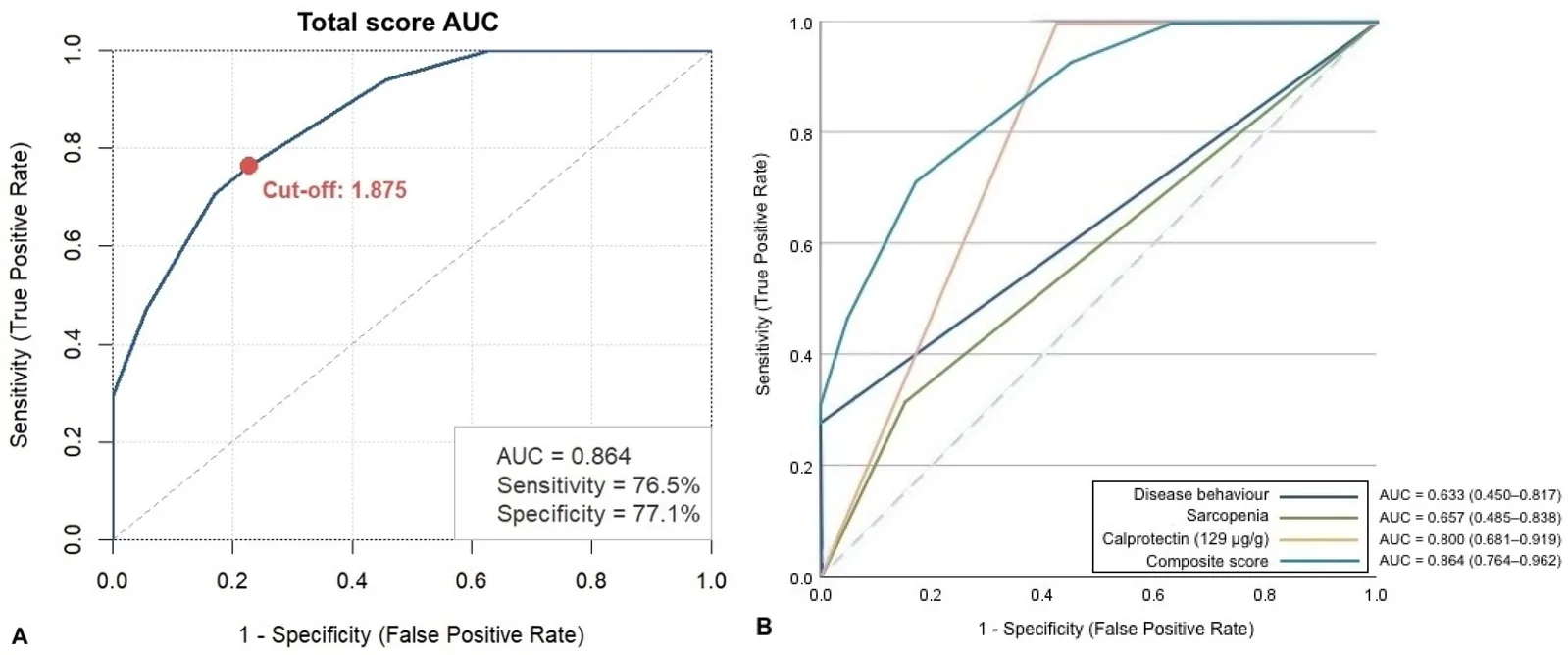
(**A**) Receiver operating characteristic (ROC) curve for the value of the proposed score in predicting disease relapse (dark blue line). (**B**) Composed graph showing the ROC curve of the score compared to those of each parameter taken separately). The 45° diagonal (interrupted line) represents the line of no discrimination (AUC = 0.5).

The composite score demonstrated superior diagnostic accuracy compared to its individual components. The apparent performance of the created clinical score showed an excellent discrimination capacity, with an area under the ROC curve (AUC) of 0.864, a Se of 76.5% and a Sp of 77.1% at the optimal threshold identified by the Youden index (1.875)—Figure 3.

In the first stage we applied the bootstrap method to estimate the precision of the AUC, Se and Sp measures (represented graphically with confidence interval) according to our proposed coefficients. The input variables and score coefficients were fixed.

**Figure 4:**
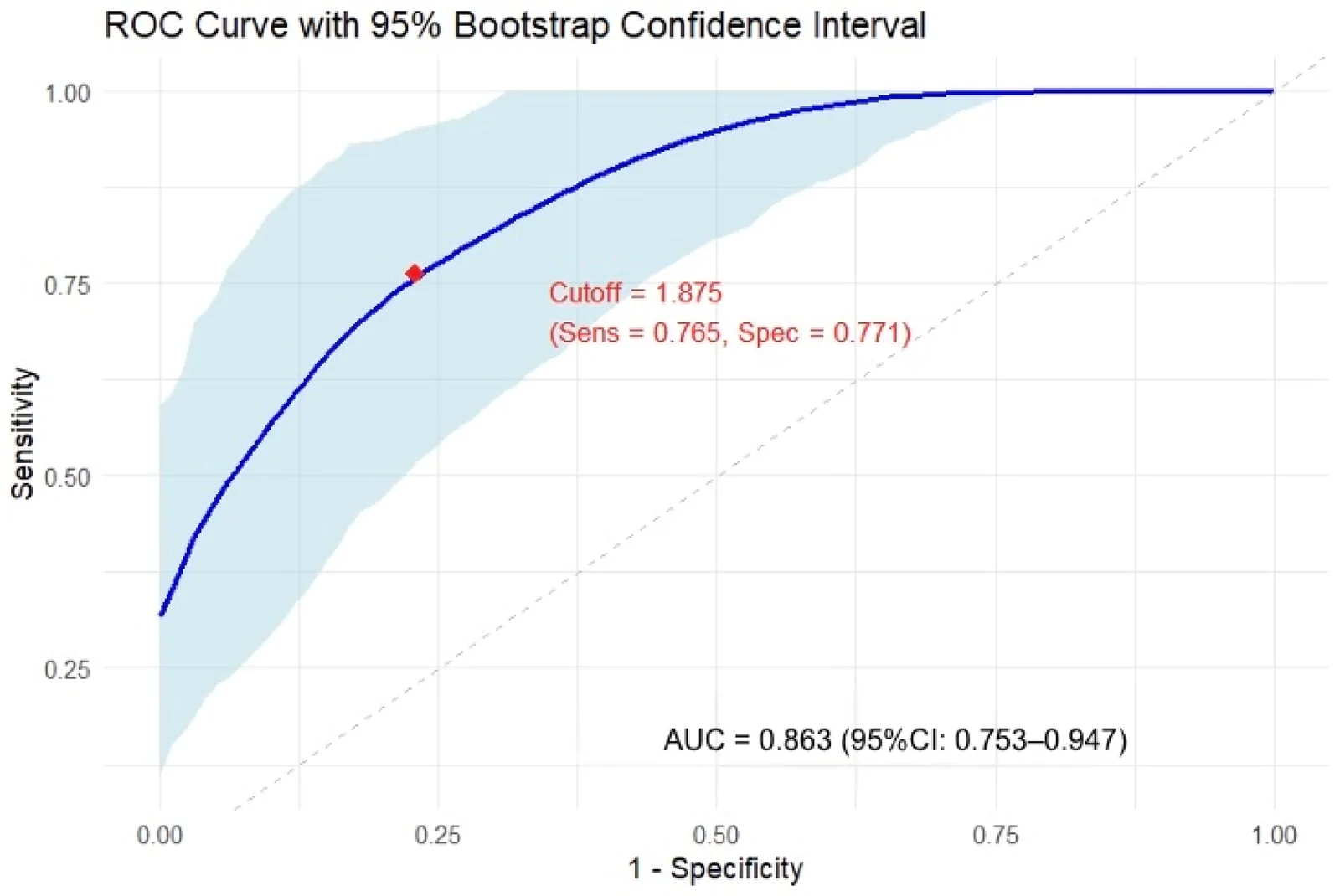
Receiver operating characteristic (ROC) curve for the composite score in predicting unfavourable outcome (UO). The curve represents mean sensitivity across 1000 bootstrap resamples at each specificity level, with the shaded band indicating the 95% bootstrap confidence interval. The diagonal dashed line represents the line of no discrimination (AUC = 0.5). The marker indicates the optimal cutoff value (score = 1.875), selected using the Youden index on the original dataset, corresponding to a sensitivity of 76.6% and specificity of 76.8%. The overall AUC was 0.863 (95% CI: 0.753–0.947), indicating good discriminative performance.

After 1000 validation samples we found a bootstrap-corrected AUC of 0.863 (Figure 4, Table 5). The bias values for Se and Sp were also small, but the standard error was larger for Se, reflecting the limited number of patients in the UOG. Consequently, the Se estimate is subject to greater sampling variability and should be interpreted with appropriate caution (Table 6).

In the second stage we fixed only the 3 input variables. Following internal validation using the bootstrap method with optimism correction [28], the degree of overfitting of the model was assessed. The coefficients deduced in this step from the original set are: Montreal disease behaviour—2.25 points; calprotectin—1.75 points; sarcopenia—1 point. The average optimism was reduced (0.025 for AUC, 0.072 for Se and 0.030 for Sp).

The final corrected score performance confirms a clinically relevant and stable discriminant capacity, with a corrected AUC of 0.839, a corrected Se of 69.2% and a corrected Sp of 74.1%. 

## 4. Discussion

Nutritional status plays an important role in patients with CD. Although multiple tools for measuring sarcopenia exist, they have certain disadvantages. For instance, clinical strength measurements are limited in patients with associated disability, and their specificity for a single muscle group only moderately correlates with muscle strength in other compartments. DXA and BIA are also accurate measurements of total muscle mass, but their results may be influenced by the hydration status and require adjustment by age, race and population standards [8]. In addition, some of these tests are expensive, not always available and require special equipment and trained specialists [29].

The composite score we propose combines sarcopenia, inflammatory status (calprotectin), and disease behaviour (Montreal B2 and B3) evaluation, which demonstrates good to great discriminative ability in predicting UO. Internal validation using bootstrap resampling (1000 iterations) confirmed the stability of these results, with a bootstrap-corrected AUC of 0.863. A score of 0 predicts sustained remission, while the presence of sarcopenia with any of the other two variables indicates a high probability (>80%) of UO. Given the limited number of events and our aim to develop a straightforward clinical scoring system, dichotomisation of continuous variables was considered an appropriate compromise despite the inevitable loss of information and statistical power. Larger external validation studies are warranted to evaluate whether retaining continuous predictors improves score performance and enables more refined risk stratification into low-, intermediate-, and high-risk groups.

Literature data has shown correlations of sarcopenia with LOR to Infliximab [6], lack of endoscopic remission, increased risk of abscess formation, and increased postoperative complications [30].Our study supports these findings, as the UOG included patients with no remission at 12 months and complications as a reason for necessitating therapeutic change or surgery.

Muscle composition analysis based on CT and MRI is considered a gold standard for sarcopenia assessment [8]. They are non-invasive tests already performed in the follow-up of CD patients, thereby enabling the evaluation of sarcopenia at no additional costs to healthcare providers. As patients with CD are mostly young and the disease follows a remissive-recurrent pattern, cumulative radiation exposure makes CT less desirable for their follow-up. However, some authors have also performed muscle mass analysis on MRI sequences with good results in predicting disease evolution [16,31,32,33]. MRI enterography is also non-irradiating and can be performed any number of times, making it a key exploration in diagnosing and staging disease in the paediatric population [21].

One study by Xie et al. [11] showed that SMI and muscle attenuation values could predict endoscopic response to biologics, an analysis that can be performed on either routine MRI for disease staging or CT performed when a complication is suspected. Muscle mass analysis by these methods showed excellent intra- and inter-rater agreement [34].

Artificial intelligence models may provide an added value, permitting the automation of the parameter extraction process and even extending the palette of parameters that can be obtained for radiomic analysis [31,35,36].

The ESPEN guideline for nutrition in inflammatory bowel diseases recommends active screening for malnutrition given the increased risk, as well as appropriate treatment to reduce the risk of complications and mortality and to improve quality of life [37]. A normal body mass index (BMI) can be present in a significant proportion of sarcopenic patients, with up to 20% of the patients in a specific cohort being overweight [38]. In our cohort, only 1 patient with sarcopenia presented a BMI in the overweight category, while 5 patients with sarcopenia presented a normal BMI. These data suggest that screening could provide additional benefits across all patient categories.

Multiple factors can be involved at the same time, with the added contribution leading to important nutritional effects: reduced food intake is the result of nausea, abdominal pain and diarrhoea from active disease or some treatment options, while malabsorption can result from restrictive diets employed during hospitalisation, accelerated intestinal transit, damaged bowel lining or bacterial overgrowth [39]. These findings support the need for direct dietary interventions, but there is substantial variability in suggested regimens, with limited guideline recommendations for a specific diet based on prospective data [40]. Enteral nutrition (EN) is an effective strategy that can be implemented during the active phase of the disease to reduce inflammation in conjunction with appropriate treatment, but its long-term use is limited in adults due to poor tolerability and no prolonged reduction in relapse or complications [40]. Other dietary regimens proposed include the CD-exclusion diet [41], the specific carbohydrate diet (SCD) [42] and the low-FODMAP (fermentable oligosaccharides, disaccharides, monosaccharides, and polyols) diet [43,44]. Supporting studies are limited, especially in SCD, while for low-FODMAP, data from small populations show only moderate effects [40].

Physical activity may benefit CD patients as well. Its effects result from reducing the pro-inflammatory cytokines and boosting the immune system, while also diminishing the risk of obesity, which is associated with a lower prevalence of remission [45]. Supplementary studies are warranted to explore the benefit of direct nutritional and physical activity interventions in CD.

Our study has several strengths. To our knowledge, this is the first study regarding sarcopenia status in patients with CD from our country, and its associations with disease relapse and incidence of complications. The proposed score demonstrated promising discrimination in this derivation cohort but requires validation in larger, independent populations. If confirmed, its application is simple and can be performed on available imaging, approved and appropriate for the evaluation of CD [46]. It also includes easily accessible independent variables, notably biological and disease behaviour parameters.

Our data align with those reported in the literature. Our statistical analysis of faecal calprotectin levels identified a cut-off value of 129 μg/g for disease relapse, consistent with values reported by other authors [47,48,49]. On the other hand, some studies report a cut-off value of 250 μg/g as more appropriate for stratifying patients [12]. This difference might be due to the small sample size of our study and represents a cohort characteristic or may be the result of the added benefit of the composed score. We also performed a univariate analysis using the 250 μg/g cutoff, which resulted in inferior predictive power (AUC = 0.638, 95% CI = 0.464–0.812). The addition of nutritional and disease behaviour parameters, which showed varying results in different studies, benefits the total predictive score by increasing the composite accuracy and providing additional value of these measurements.

Our study also has several limitations, the most important of which is the small population size, particularly the small number of patients with UO. Because multiple logistic regression models require an adequate number of outcome events to ensure stable coefficient estimates, our model may be susceptible to overfitting. In the current form, the number of EPV is at 5.7, below the commonly recommended thresholds. Consequently, the observed AUC may overestimate the model’s predictive performance and external validation in larger, independent cohorts is required before any clinical application.

Under these conditions, internal validation via a 1000-replication nonparametric bootstrap was vital to prevent overoptimistic conclusions and to assess model reproducibility. The bootstrap-estimated AUC was 0.863 (95% CI: 0.753–0.948), with minimal bias (−0.0006), suggesting the score’s discriminative performance is unlikely to be substantially inflated by overfitting despite the limited sample size. Sensitivity and specificity remained consistent across resamples, with bootstrap means of 76.7% (95% CI: 53.8–94.4%) and 76.9% (95% CI: 61.8–89.7%) respectively. The relatively wide confidence intervals—particularly for sensitivity—reflect the limited sample size (*n* = 52) and should be interpreted with appropriate caution. Nonetheless, the narrow bias observed in the bootstrap estimates, combined with an AUC confidence interval that remains well above the 0.5 threshold of no discrimination supports the validity of the suggested score. The optimism corrected AUC remains as well above the values of individual parameters, at 0.839, with good Se and Sp.

Similarly, the inclusion of patients from a single tertiary centre, treated only with biologics and small molecules, could have induced patient selection bias. Our decision was based on the fact that prolonged steroid treatment is associated with muscle wasting [50,51,52] and could significantly influence the results. In addition, there is high variability in the presentation of CD patients in our hospital, and our study cohort included patients of different ages with both long-standing and relatively short disease evolution, ensuring a broad range of variability. Due to the retrospective nature, the clinical, biological, imaging and endoscopic evaluations were performed according to routine clinical practice rather than using a predefined protocol. By excluding from the study the patients with incomplete datasets, we aimed to reduce the heterogeneity and improve the ability to compare the outcomes. Due to all these biases, although the score demonstrated a promising predictive performance, external validation is mandatory before being generalised to the broader Crohn’s disease population.

The primary endpoint was defined as a composite of relapse, need for treatment escalation, surgery, and radiological or endoscopic evidence of disease activity, which suggests a high heterogeneity. Their weight is supported by a difference in their clinical severity and implications for patient management, limiting the ability to draw conclusions in predicting each individual component. The relatively small sample size precluded separate analyses of the individual outcome components, supporting the need for future studies with larger cohorts to evaluate the predictive performance of the score for each clinically relevant endpoint separately.

The behaviour item of the Montreal classification included in our proposed score also restricts its applicability in patients with UC. This is an important difference as CD is a transmural process in most cases, which can lead to stenosis and penetrating disease (classified as Montreal B2 and B3).

## 5. Conclusions

We conducted an exploratory study to assess the added role of sarcopenia in stratifying patients with CD. The proposed score offers a multimodal approach to predicting therapeutic failure in CD, obtaining a diagnostic accuracy (AUROC 0.863) that outperforms individual biomarkers. Internal validation increases confidence in the stability of our score; however, validation in external cohorts is required before clinical application can be considered. Confirmation from extensive studies and in prospective interventional studies can strengthen the role of imaging by adding sarcopenia as a predictive factor for disease progression.

## Figures and Tables

**Table 1:** Patient demographics. Age and disease duration are shown as mean ± standard deviation.

No. of Patients	52
F:M	25:27
Age (years)	46.49 ± 18.28
Body mass index	21.53 ± 3.14
Tobacco use	2
Disease duration (years)	5.58 ± 4.66
Treatment	IFX = 13
	ADA = 19
	UST = 12
	UPA = 3
	VEDO = 5
Previous treatment changes	11
CD-related surgery in the past	19
Previous complicated disease	7

ADA = Adalimumab; CD = Crohn’s disease; IFX = Infliximab; F:M = female-to-male ratio; UPA = Upadacitinib; UST = Ustekinumab; VEDO = Vedolizumab.

**Table 2:** Analysis of the parameters that correlated with unfavourable outcomes.

Independent Variable	FOG (*n* = 35)		UOG (*n* = 17)		*p*
	Mean	SD	Mean	SD	
**Nutritional status parameters**					
Skeletal muscle area (SMA)	131.28	33.31	120.57	32.5	0.04
Skeletal muscle index (SMI)	46.98	9.37	42.43	10.07	<0.001
Sarcopenia (*n* = 19)	7 cases		12 cases		0.036
Hemoglobin	12.29	1.32	11.56	2.01	0.138
Albumin	4.117	0.57	3.87	0.60	0.672
**Biologic parameters**					
C-reactive protein	2.86	4.04	6.34	6.70	0.02
Calprotectine	955	1490.64	276.09	393.78	<0.001
**Disease behaviour parameters**					
Disease behaviour phenotype	B1	-	B2-B3	-	0.003
Previous treatment failure	No	-	Yes	-	0.01

FOG = favourable outcome group; SD = standard deviation; UOG = unfavourable outcome group.

**Table 3:** The parameters identified that showed the highest predictive value for an unfavourable outcome.

Item	B	S.E.	Wald	df	Sig.	Exp(B)	95% C.I. for EXP (B)	
							Lower	Upper
Disease behaviour (B phenotypes)	2.337	0.917	6.495	1	0.011	10.347	1.715	62.415
Calprotectin (>129 μg/g)	2.143	0.887	5.830	1	0.016	8.522	1.497	48.522
Sarcopenia	1.582	0.821	3.715	1	0.054	4.863	0.974	24.289
Constant	−3.495	0.945	13.668	1	0.000	0.030		

Exp (B) Odds Ratio. B—weight. CI—confidence interval. The probability of risk (*p*) was calculated using the formula *p* = 1/(1 + e − (−3.495 + 2.143 × X1 +2.337 × X2 +1.582 × X3) where X1, X2, and X3 are binary variables (1 = present, 0 = absent) representing Calprotectin >129 μg/g, complicated disease behavior (Montreal B2/B3), and Sarcopenia, respectively.

**Table 4:** The resulting score is derived from multivariate analysis, weighted by the strength of each parameter.

Parameter	Cutoff	Score Value
Behaviour type	B1	0 points
	B2-B3	2 points
Calprotectin	<129 μg/g	0 points
	>129 μg/g	1.75 points
Sarcopenia	no sarcopenia	0 points
	sarcopenia	1 point

**Table 5:** Bias calculated after bootstrap testing of the proposed score.

Parameter	Original Testing	Bias After Bootstrap Testing	Standard Error
AUC	0.864	−0.0006	0.049
Sensitivity	0.764	+0.0018	0.105
Specificity	0.771	−0.0028	0.071

AUC = area under the curve.

**Table 6:** The bootstrap mean values and their confidence interval.

Parameter	Mean	95% Confidence Interval
AUC	0.863	0.753–0.947
Sensitivity	0.766	0.538–0.944
Specificity	0.768	0.617–0.897

AUC = area under the curve.

## Data Availability

The datasets analysed during the current study are not publicly available due to patient privacy and institutional restrictions but are available from the corresponding author on reasonable request and with the permission of the institution.

## Supplementary Materials

The following supporting information can be downloaded at https://www.mdpi.com/article/10.3390/biomedicines14081855/s1. Table S1: STROBE Statement—checklist of items that should be included in reports of observational studies; Table S2: The Hosmer-Lemeshow Contingency table and test.

## Abbreviations

ADA = adalimumab; AUC = area under the curve; BIA = bioelectrical impedance analysis; CD = Crohn’s disease; CDAI = Crohn’s disease activity index; CRP = C-reactive protein; DXA = dual X-ray absorptiometry; FOG = favourable outcome group; HU = Hounsfield units; IBD = inflammatory bowel disease; IFX = infliximab; IL = interleukin; IQR = interquartile range; SD = standard deviation; SMA = skeletal muscle area; SMI = skeletal muscle index; TNF = tumour necrosis factor; UC = ulcerative colitis; UO = unfavourable disease outcome; UOG = unfavourable outcome group; UPA = Upadacitinib; UST = Ustekinumab; VDO = Vedolizumab.
