# Supplementary material for: Beyond Inflammation: Sarcopenia and Disease Phenotype Predict Unfavourable Course in Crohn’s Disease—A Retrospective Observational Study
Source: Biomedicines. 2026 Aug 18;14(8):1855. doi: 10.3390/biomedicines14081855 (PMC13509676; doi:10.3390/biomedicines14081855)
Supplement: Supplementary file 1 [file biomedicines-14-01855-s001.zip › biomedicines-4333217-supplementary.pdf]

Supplementary Table S1. STROBE Statement—checklist of items that should be included in reports of observational studies

	Item No.	Recommendation	Page No.	Relevant text from manuscript
**Title and abstract**	1	(*a*) Indicate the study's design with a commonly used term in the title or the abstract	title page	A Retrospective Observational Study
		(*b*) Provide in the abstract an informative and balanced summary of what was done and what was found	1	We conducted a retrospective study of patients with Crohn's disease […] hospitalized at our institution between January 2020 and June 2024. Using previous CT scans, we measured skeletal muscle mass (SMM) and skeletal muscle index (SMI) to assess their association with disease outcomes.
**Introduction**				
Background/rationale	2	Explain the scientific background and rationale for the investigation being reported	3	While the correlation between sarcopenia and unfavourable disease outcomes (UO) […] is well documented, its predictive value is likely maximized when integrated into comprehensive multivariate scoring systems rather than used as an isolated marker.
Objectives	3	State specific objectives, including any prespecified hypotheses	4	We aim to reduce the knowledge gap regarding factors that may predict UO in CD patients by analysing multiple categories of factors, including sarcopenia and inflammatory status markers.
**Methods**				
Study design	4	Present key elements of study design early in the paper	4	This retrospective observational study included patients with CD followed up in the Gastroenterology Department of the "Sf. Spiridon" Emergency Clinical County Hospital, Iași, Romania.
Setting	5	Describe the setting, locations, and relevant dates, including periods of recruitment, exposure, follow-up, and data collection	5	We included patients with a confirmed diagnosis of CD who were hospitalized in our department between January 2020 and June 2024.
Participants	6	(*a*) *Cohort study*—Give the eligibility criteria, and the sources and methods of selection of participants. Describe methods of follow-up	5	The inclusion criteria were: adult patients with confirmed CD according to the ECCO-ESGAR Guideline for Diagnostic Assessment of IBD undergoing biologic or small molecule treatment, in which a CT examination and blood tests were

		Case-control study—Give the eligibility criteria, and the sources and methods of case ascertainment and control selection. Give the rationale for the choice of cases and controls<br>*Cross-sectional study*—Give the eligibility criteria, and the sources and methods of selection of participants		performed in the same week, were available for data collection. We included only patients who had at least one follow-up visit between 6 and 12 months after baseline imaging. The exclusion criteria included: patients under 18 years; lack of imaging or blood test data; corticoid therapy; lack of sequential follow-up visits; other chronic diseases that may lead to patient wasting (e.g., malignancies, congestive heart failure, chronic kidney disease, neuromuscular disease); and pregnancy.
		(*b*) *Cohort study*—For matched studies, give matching criteria and number of exposed and unexposed<br>*Case-control study*—For matched studies, give matching criteria and the number of controls per case		N/A
Variables	7	Clearly define all outcomes, exposures, predictors, potential confounders, and effect modifiers. Give diagnostic criteria, if applicable	6-7	Based on the data obtained from patient medical records, we created two outcome groups:<br>- Patients with sustained clinical (according to the Crohn's disease activity index - CDAI) and biologic (normalisation or reduction by >50% of the initial CRP value +/- calprotectin levels when available) remission at the follow-up visits, in whom the colonoscopy and/or imaging (either CT or MRI) showed no acute bowel inflammation were considered sustained responders and added to the favourable outcome group (FOG);<br>- Patients in whom the disease evolution was marked by relapse, the need for changing the therapeutic line or addition of corticotherapy, surgery, as well as endoscopic and/or imaging findings suggestive of active disease, were included in the unfavourable outcome group after ruling out other conditions that may simulate active IBD (UOG).

Data sources/ measurement	8*	For each variable of interest, give sources of data and details of methods of assessment (measurement). Describe comparability of assessment methods if there is more than one group	5<br>6	Data were extracted from medical records using a standardized collection sheet.<br>The skeletal mass area (SMA) and skeletal mass index (SMI) at the level of the L3 vertebral body were extracted from the available CT images using 3D slicer […]
Bias	9	Describe any efforts to address potential sources of bias	5	The exclusion criteria included: […] corticoid therapy; […] other chronic diseases that may lead to patient wasting (e.g., malignancies, congestive heart failure, chronic kidney disease, neuromuscular disease); and pregnancy.
Study size	10	Explain how the study size was arrived at		N/A

Continued on next page

Quantitative variables	11	Explain how quantitative variables were handled in the analyses. If applicable, describe which groupings were chosen and why	7	Data distribution normality was assessed using the Shapiro-Wilk test. Continuous variables were expressed as mean ± standard deviation (SD) for normally distributed data, or as median with interquartile range (IQR) for non-normally distributed data. Categorical variables were presented as frequencies and percentages.
Statistical methods	12	(*a*) Describe all statistical methods, including those used to control for confounding	7	[…] Data distribution normality was assessed using the Shapiro-Wilk test.<br>To determine the association between study variables and disease outcomes, we used parametric tests (independent-samples t-test) for normally distributed continuous variables and nonparametric tests (Mann-Whitney U test) for those with nonnormal distributions. Categorical variables were compared using the Chi-square test or Fisher's exact test, as appropriate. Bivariate analysis was conducted to assess correlations between physiological parameters (e.g., SMM and SMI) using Pearson or Spearman analysis as appropriate.<br>To further assess the influence of study variables on therapeutic efficacy duration, we performed a Cox proportional hazards regression analysis. […]<br>Univariate and multivariate binary logistic regression analyses were performed to identify independent predictors of UO. Finally, Receiver Operating Characteristic (ROC) curve analysis was used to calculate the Area Under the Curve (AUC) and to determine optimal cut-off values for constructing the predictive scoring system. […]
		(*b*) Describe any methods used to examine subgroups and interactions		To further assess the influence of study variables on therapeutic efficacy duration, we performed a Cox proportional hazards regression analysis.
		(*c*) Explain how missing data were addressed		N/A

		(*d*) *Cohort study*—If applicable, explain how loss to follow-up was addressed<br>*Case-control study*—If applicable, explain how matching of cases and controls was addressed<br>*Cross-sectional study*—If applicable, describe analytical methods taking account of sampling strategy		N/A
		(*e*) Describe any sensitivity analyses		N/A
**Results**				
Participants	13*	(a) Report numbers of individuals at each stage of study—eg numbers potentially eligible, examined for eligibility, confirmed eligible, included in the study, completing follow-up, and analysed	8	The initial search of the prospectively maintained database yielded 231 patients with CD in whom imaging was performed to evaluate suspected disease-associated complications (n=109), disease staging (n=43), or unrelated pathologies (n=79) such as trauma or acute abdomen. After applying the exclusion criteria, we were left with 50 patients (54% male) […]
		(b) Give reasons for non-participation at each stage	9	Patient selection flowchart
		(c) Consider use of a flow diagram	9	Figure 1
Descriptive data	14*	(a) Give characteristics of study participants (eg demographic, clinical, social) and information on exposures and potential confounders	8	Table 1
		(b) Indicate number of participants with missing data for each variable of interest		N/A
		(c) *Cohort study*—Summarise follow-up time (eg, average and total amount)		N/A
Outcome data	15*	*Cohort study*—Report numbers of outcome events or summary measures over time	10	Table 2 (favourable outcome group = 35 patients, unfavourable outcome group = 15 patients)
		Case-control study—Report numbers in each exposure category, or summary measures of exposure		N/A
		Cross-sectional study—Report numbers of outcome events or summary measures		N/A
Main results	16	(*a*) Give unadjusted estimates and, if applicable, confounder-adjusted estimates and their precision (eg, 95% confidence		N/A

	interval). Make clear which confounders were adjusted for and why they were included		
	(*b*) Report category boundaries when continuous variables were categorized	N/A	
	(*c*) If relevant, consider translating estimates of relative risk into absolute risk for a meaningful time period	N/A	

Continued on next page

Other analyses	17	Report other analyses done—eg analyses of subgroups and interactions, and sensitivity analyses	13	Table 4 (multivariate analysis lead to the proposal of a score, with an AUROC of 0.863 – Figure 3)
**Discussion**				
Key results	18	Summarise key results with reference to study objectives	14	In our analysis, its addition to the inflammatory and behaviour parameters provides a simple, non-invasive tool that effectively stratifies patients' outcomes: a score of 0 predicts sustained remission, while the presence of elevated calprotectin combined with either sarcopenia or complicated behaviour indicates a high probability (>80%) of UO, warranting closer monitoring or therapeutic optimization.
Limitations	19	Discuss limitations of the study, taking into account sources of potential bias or imprecision. Discuss both direction and magnitude of any potential bias	16	Our study also has several limitations. Firstly, the retrospective nature […]. The inclusion of patients from a single centre could have induced patient selection bias, however there is a high variability in the presentation of CD patients in our hospital. We did not perform the analysis on patients with UC, and the behaviour subgroup of the Montreal classification that was included in our score could not be applied in these patients.
Interpretation	20	Give a cautious overall interpretation of results considering objectives, limitations, multiplicity of analyses, results from similar studies, and other relevant evidence	15	Similar studies have shown correlations of sarcopenia with LOR to infliximab, lack of endoscopic remission, increased risk of abscess formation, as well as increased postoperative complications.
			15	As the patients with IBD are mostly young, and due to the remissive-recurrent pattern, the compound radiation exposure makes CT less desirable for their follow-up. However, some authors have also performed muscle mass analysis on MRI sequences with good results in predicting disease evolution.
			15	Muscle mass analysis by these methods showed excellent intra- and inter-rater agreement.
Generalisability	21	Discuss the generalisability (external validity) of the study results	17	The composite scoring system developed in this study represents a robust, multimodal approach to predicting therapeutic failure in IBD, obtaining a diagnostic accuracy (AUROC 0.863) that significantly outperforms individual biomarkers.
**Other information**				

Funding	22	Give the source of funding and the role of the funders for the present study and, if applicable, for the original study on which the present article is based	4/declarations	This research was funded by The Health Programme (PS) 2021-2027, Policy Objective 4, Priority 3, Project title "Support for Enhancing Performance and Innovation in Excellent Medical Doctoral Research", acronym INNODOC-MED, SMIS code 351058).

*Give information separately for cases and controls in case-control studies and, if applicable, for exposed and unexposed groups in cohort and cross-sectional studies.

Note: An Explanation and Elaboration article discusses each checklist item and gives methodological background and published examples of transparent reporting. The STROBE checklist is best used in conjunction with this article (freely available on the Web sites of PLoS Medicine at http://www.plosmedicine.org/, Annals of Internal Medicine at http://www.annals.org/, and Epidemiology at http://www.epidem.com/). Information on the STROBE Initiative is available at www.strobe-statement.org.

Supplementary Table S2. The Hosmer-Lemeshow Contingency table and test

Contingency Table for Hosmer and Lemeshow Test

		LOR = 0		LOR = 1		
		Observed	Expected	Observed	Expected	Total
Step 1	1	13	12.617	0	.383	13
	2	6	6.100	1	.900	7
	3	8	8.740	3	2.260	11
	4	2	2.283	1	.717	3
	5	4	3.543	4	4.457	8
	6	2	1.360	3	3.640	5
	7	0	.357	5	4.643	5

Hosmer and Lemeshow Test

Step	Chi-square	df	**Sig.**
1	1.762	5	**.881**
